# Gemcitabine-induced neutrophil extracellular traps via interleukin-8-CXCR1/2 pathway promote chemoresistance in pancreatic cancer

**DOI:** 10.1038/s41416-025-03192-1

**Published:** 2025-09-25

**Authors:** Shohei Nogi, Shunsuke Kagawa, Atsuki Taniguchi, Tomohiko Yagi, Nobuhiko Kanaya, Yoshihiko Kakiuchi, Kazuya Yasui, Tomokazu Fuji, Yoshiyasu Kono, Satoru Kikuchi, Kosei Takagi, Shinji Kuroda, Fuminori Teraishi, Hiroshi Tazawa, Toshiyoshi Fujiwara

**Affiliations:** 1 Department of Gastroenterological Surgery, Okayama University Graduate School of Medicine, Dentistry and Pharmaceutical Sciences, Okayama, Japan; 2 Center for Clinical Oncology, Okayama University Hospital, Okayama, Japan; 3 Minimally Invasive Therapy Center, Okayama University Hospital, Okayama, Japan; 4 Organ Transplant Center, Okayama University Hospital, Okayama, Japan; 5 Department of Gastroenterology and Hepatology, Faculty of Medicine, Dentistry and Pharmaceutical Sciences, Okayama University, Okayama, Japan; 6 Center for Innovative Clinical Medicine, Okayama University Hospital, Okayama, Japan

**Keywords:** Cancer microenvironment, Pancreatic cancer

## Abstract

**Background:**

Pancreatic ductal adenocarcinoma (PDAC) is one of the most aggressive cancers, and chemoresistance poses a significant challenge in its treatment. Neutrophil extracellular traps (NETs) have emerged as key players in the tumour microenvironment, but their role in chemoresistance remains unclear.

**Methods:**

We investigated the involvement of NETs in PDAC chemoresistance using patient tumour samples, in vitro assays with gemcitabine (GEM)-treated PDAC cells, and in vivo mouse models. We evaluated cytokine production, NET formation and tumour response to GEM, with or without the CXCR1/2 inhibitor navarixin.

**Results:**

NETs are significantly accumulated in the tumours of PDAC patients exhibiting poor response to chemotherapy. GEM-treated PDAC cells secrete pro-inflammatory cytokines such as interleukin-8 (IL-8). IL-8 promote the formation of chemotherapy-induced NETs (chemoNETosis) through activation of CXCR 1/2 on neutrophils. Importantly, treatment with navarixin significantly suppressed chemoNETosis, restored sensitivity to GEM, and significantly reduced tumour growth in vivo.

**Conclusions:**

Our findings reveal that NETs contribute to chemoresistance in PDAC and that IL-8–mediated chemoNETosis plays a pivotal role in this process. Inhibition of CXCR1/2-mediated NET formation enhances the efficacy of GEM. This approach may represent a promising therapeutic strategy for overcoming chemoresistance in PDAC. These results support further clinical investigation of anti-NETs therapies.

## Background

Pancreatic ductal adenocarcinoma (PDAC) is one of the most lethal malignancies, ranking 4th in cancer-related mortality among men and 3rd among women in the United States [1]. Despite advancements in treatment, early diagnosis remains challenging and more than 80% of PDAC cases are diagnosed at an advanced stage [2]. Chemotherapy is the standard treatment for advanced PDAC. However, resistance due to various factors intrinsic to cancer cells, cancer stem cells and the tumour microenvironment (TME) poses a significant challenge [3, 4].

Neutrophils within the TME are one of the immune cell subsets most strongly associated with poor prognosis in various cancers [5]. Tumour-associated neutrophils have also been implicated in poor outcomes and reduced chemotherapy efficacy in PDAC [6–9]. Among the tumour-promoting mechanisms of neutrophils, the formation of neutrophil extracellular traps (NETs) is well-recognised [10–12]. NETs are large, extracellular, web-like structures composed of cytosolic and granule proteins that are assembled on a scaffold of decondensed chromatin. They were originally identified as a defence mechanism against bacterial infections [13]. Our research group has demonstrated that NETs induce epithelial-to-mesenchymal transition (EMT) in PDAC cells and promote metastasis [14].

Furthermore, NETs release immunosuppressive factors and inhibit the interaction between tumours and immune cells, thereby creating an immunosuppressive microenvironment [11]. Consequently, the inhibition of NETs has been shown to enhance the efficacy of immunotherapy [15–17].

Additionally, NETs have been reported as predictive markers for chemotherapy efficacy in locally advanced rectal cancers and metastatic renal cell carcinoma [18, 19], suggesting that NETs are also associated with chemotherapy efficacy. Recently, chemotherapy-induced NETs (chemoNETosis) was reported for the first time in breast cancer, where it contributed to chemoresistance via TGF-β activation [20]. However, the effect of NETs on cytotoxic chemotherapy in the other cancers remains largely unexplored. We hypothesised that a similar phenomenon might occur in PDAC. In this setting, NETs could induce chemoresistance and impair the effectiveness of chemotherapy. We conducted experiments to investigate this hypothesis.

## Methods

### Cell culture and reagents

The human PDAC cell lines MIAPaCa-2 and BxPC3 were obtained from the American Type Culture Collection, and KP4 was obtained from the Japanese Collection of Research Bioresources Cell Bank. The mouse PDAC cell line PAN02 was obtained from the U.S. National Cancer Institute. Gemcitabine-resistant (GR) PAN02 cells were established previously [21], and the methods for their establishment are described in Supplementary Materials and Methods. Gemcitabine (GEM); phorbol 12-myristate 13-acetate (PMA); the peptidyl arginine deiminase 4 inhibitor (PAD4i), GSK484; deoxyribonuclease (DNase) I; C-X-C chemokine receptor (CXCR) inhibitor, navarixin and reparixin; Interleukin-8 (IL-8) was used as described in Supplementary Material and Methods.

### Patients and tissue samples

Immunofluorescence staining for NETs was performed on primary tumour tissues from PDAC patients who underwent surgery after neoadjuvant chemotherapy (NAC) with the GEM plus nab-paclitaxel (GN) regimen at Okayama University Hospital between September 1, 2015, and September 30, 2021. All samples were collected from patients who provided informed consent and all related procedures were performed with the approval of the internal review and ethics board of Okayama University Hospital (No. 2406-025).

### In vitro NETosis assay

Neutrophils (5 × 10^5^ cells/well) were seeded on inner cover slips (#C018001, Matsunami, Japan) in a 12-well plate (#353043, Falcon) and pretreated with GSK484, DNase I, navarixin, or reparixin for 30 min. Subsequently, they were treated with PMA or Cancer conditioned media (CM) for 6 h with the previously mentioned reagents. The cover slips were retrieved and fixed with 4% paraformaldehyde, and after staining the extracellular DNA with SYTOX Green (25 nM, #S7020, Thermo Fisher) for 15 min, they were observed with IX83 microscope (Olympus, Tokyo, Japan). Only structures depicting NETs morphology and positive for SYTOX Green were selected for area quantification, and intact granulocyte nuclei were excluded from the analysis.

### In vivo experiments

Animal experimental protocols were approved by the Ethics Review Committee for Animal Experimentation of the Okayama University School of Medicine (No. OKU-2023195). Parental and GR PAN02 cells (4 × 10^6^ cells) were subcutaneously inoculated into the flanks of 6-week-old female C57BL/6 J mice (CLEA Japan, Tokyo, Japan). MIAPaCa-2 cells (5 × 10^6^ cells) were subcutaneously inoculated into the flanks of 6-week-old female BALB/c-nu/nu mice (CLEA Japan, Tokyo, Japan). In the PAN02 subcutaneous tumour model, treatment was started on the 7th day after tumour inoculation, when it became palpable. Mice were injected intraperitoneally twice a week for 5 weeks with 100-µL volume of phosphate-buffered saline (PBS) or a solution containing GEM (100 mg/kg). In the MIAPaCa-2 subcutaneous tumour model, treatment was initiated on the 10th day after tumour inoculation. In the low-dose GEM treatment, 30 mg/kg was administered, and in the final combination treatment experiment, GEM was administered at 50 mg/kg and navarixin at 3 mg/kg via intraperitoneal injection twice a week for 5 weeks. The tumour volume was calculated using the following formula: tumour volume (mm^3^) = a × b^2^ × 0.5, where a represents the largest diameter, b represents the smallest diameter and 0.5 is a constant used to calculate the volume of an ellipsoid.

### Public dataset analysis

The Gene Expression Omnibus (GEO) dataset GSE223303 was used to explore the effects of GEM treatment on PDAC cells. Differential expression genes (DEGs) analysis was performed using the ‘edgeR, ver. 4.2.0’ in R, ver. 4.4.0 package, with screening criteria of *P* < 0.05, and |log2FC| ≥ 1. ‘ClusterProfiler, ver. 4.12.0’ in R package was used for Gene Ontology (GO) enrichment and gene set enrichment analysis (GSEA).

### In vitro induction of chemoresistance assay

Cells were seeded in 96-well plates at a density of 2 × 10^3^ cells/well (MIAPaCa-2 and KP4) or 3 × 10^3^ cells/well (BxPC-3). After 24 h, the cells were treated with GEM in the absence of foetal bovine serum, and cytotoxicity was compared with and without the addition of NETs-CM or co-culture with 1 × 10^4^ neutrophils per well. Cell viability was determined 72 h after treatment using a TACS® XTT Cell Proliferation Assay (R&D Systems, Minneapolis, MN, USA), according to the manufacturer’s protocol. The ratio of cell density reduction due to GEM treatment to the untreated cell density for each condition was compared to determine whether chemoresistance was induced.

### Apoptosis assay

Cellular apoptosis was quantified by flow cytometry using an FITC Annexin V Apoptosis Detection Kit with PI (#640914, BioLegend) according to the manufacturer’s instructions, with slight modifications. Briefly, cells were seeded in 6-well plates at a density of 4 × 10^4^ cells/well (MIAPaCa-2 and KP4) or 6 × 10^4^ cells/well (BxPC-3). After 24 h, the cells were treated with GEM with or without NETs-CM for 72 h, harvested and suspended in 100 μL of Annexin V Binding Buffer containing 3 μL Annexin V-FITC and 6 μL PI. The cells were incubated for 15 min at room temperature in the dark. The cell suspension was diluted to 500 μL with Annexin V binding buffer, and the apoptotic cells were detected using a BD FACSLyric™ flow cytometer (BD Biosciences, San Jose, CA, USA). Data were analysed using FlowJo software, ver. 10.6.1.

### Statistical analysis

All statistical analyses and graphical representations were performed using the GraphPad Prism 10 software. Comparisons between two groups were conducted using Student’s *t-*test. For comparisons among multiple groups, one-way analysis of variance (ANOVA) followed by Tukey’s post hoc test was applied. All data were analysed using two-tailed tests, and statistical significance was defined as follows: *P < 0.05, **P < 0.01, ***P < 0.001 and ****P < 0.0001.

Details of materials and methods are available in the supplementary material.

## Results

### NETs are common in PDAC with poor NAC response

**Fig. 1:**
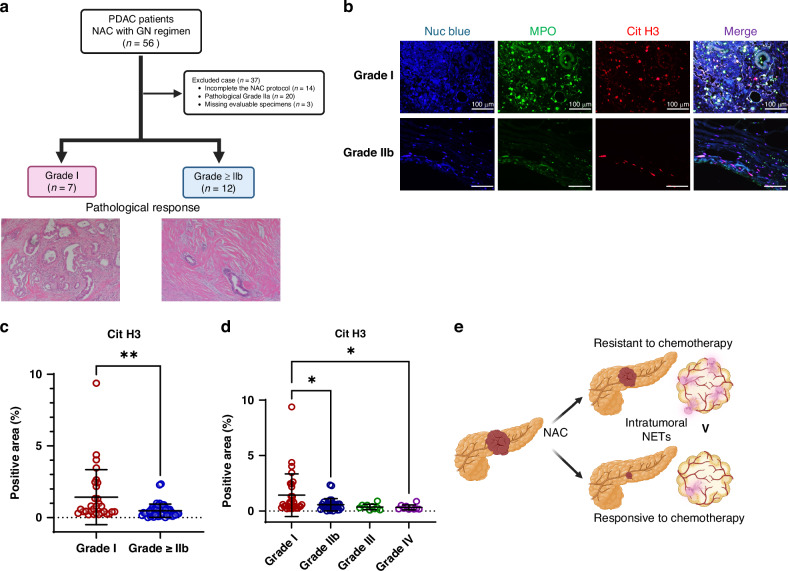
NETs are common in PDAC with poor NAC response. **a** Flowchart showing the eligibility criteria for clinical specimens and HE staining images of typical tumours. **b**–**d** Differences in the abundance of NETs by treatment response, as demonstrated by multiple IF staining for MPO and CitH3, in PDAC patients. **b** Representative fluorescence image (scale bar: 100 μm). **c**, **d** Statistical evaluation of NETs by treatment effect based on CitH3-positive area. **e** Schematic diagram showing the relationship between the response to chemotherapy for PDAC and intratumoral NETs. *P < 0.05; **P < 0.01. Data were presented as individual values and median.

To investigate the correlation between the efficacy of chemotherapy and the presence of NETs in PDAC patients, tissue samples from patients who underwent resection after NAC were categorised into responder and non-responder groups based on the Evans regression grading system [22]. Responders were defined as those with Evans grade IIb or higher, whereas non-responders were defined as those with Evans grade I (Fig. 1a). The non-responder group had significantly more R1 resections, whereas age, sex and pre-treatment tumour size showed no significant differences (Table S1). Multicolour immunofluorescence staining for myeloperoxidase (MPO) and citrullinated histone H3 (CitH3) was performed to detect NETs in paraffin-embedded sections. The results showed significantly higher accumulation of intratumoral NETs in the non-responder group than in the responder group (Fig. 1b–d). These findings suggest an inverse correlation between chemotherapy efficacy and intratumoral NETs density (Fig. 1e). However, it remains unclear whether the high NETs density caused a poor chemotherapy response or if the poor response led to increased NETs accumulation.

### GEM-treated PDAC cells induce chemoNETosis

**Fig. 2:**
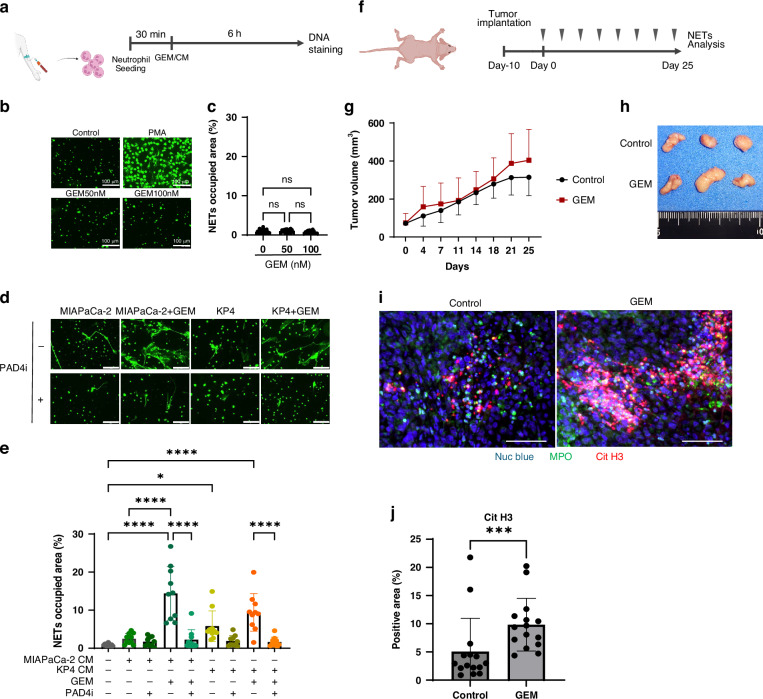
GEM-treated PDAC cells induce ChemoNETosis. **a** Outline of NETosis assay protocol in vitro. The DNA released as NETs were evaluated using SYTOX Green staining. **b** DNA staining image when adding GEM or PMA (scale bar: 100 μm). **c** Statistical evaluation of NETs by GEM stimulation based on extracellular DNA occupied area. **d** DNA staining image when adding CM from PDAC cells (scale bar: 100 μm). **e** Statistical evaluation of NETs by CM from PDAC cells. CM from GEM-treated PDAC cells causes strong NETs and PAD4i suppressed it. **f** In vivo experiment protocol for mouse subcutaneous tumour model (3 mice/group). Mice were injected intraperitoneally twice a week for 4 weeks with a 100-µL volume of PBS or low dose GEM (30 mg/kg). **g** Tumour growth curves for non-treated control (black line) and GEM-treated (red line) mice. **h** Photographs of tumours in control and GEM-treated groups. **i** Immunofluorescence staining of Nuc blue (blue), MPO (green) and CitH3 (red) in the tumour of each condition (scale bar: 100 μm). **j** Statistical evaluation of NETs based on CitH3-positive area. *P < 0.05; **P < 0.01; ***P < 0.001; ****P < 0.0001; ns no significance. Data were presented as means ± SD.

To examine whether chemotherapy promotes NETs formation in PDAC, human neutrophils from peripheral blood were subjected to NETs formation following stimulation with GEM or cancer cell CM (Fig. 2a). Direct GEM treatment did not significantly induce NETs (Fig. 2b, c). In contrast, the CM from PDAC cells stimulates neutrophils to form NETs [14]. Surprisingly, the CM from GEM-treated PDAC cells significantly enhanced NETs induction. This effect was suppressed by the addition of the PAD4i (Fig. 2d, e). Similarly, we conducted NETs induction experiments using neutrophils extracted from mouse bone marrow and CM from PAN02 cells. GEM alone did not induce NETs (Fig. S1A, B), but CM from GR PAN02 cells enhanced NETs formation compared to parental CM (Fig. S1C, D). To evaluate NETs in vivo, a subcutaneous PDAC model was established and treated with GEM (Fig. 2f). Low-dose GEM treatment did not reduce the size of the MIAPaCa-2 subcutaneous tumours (Fig. 2g, h). Despite the lack of significant differences in tumour volume, multicolour immunofluorescence staining revealed significantly increased NETs accumulation in tumours in the GEM-treated group (Fig. 2i, j). In similar experiments using parental and GR PAN02 cells (Fig. S1E), GEM treatment reduced the size of tumours derived from parental cells (Fig. S1F, G), and NETs accumulation tended to increase in the GEM-treated groups. Compared to the untreated and GEM-treated groups, GR tumours exhibited a trend toward increased NETs formation. Significantly more NETs accumulated in GR tumours treated with GEM than in untreated parental tumours (Fig. S1H, I). These findings indicate that the treatment of PDAC cells with GEM enhances NETs formation, suggesting the occurrence of chemoNETosis in PDAC.

### GEM-treated PDAC cells secrete more inflammatory cytokines

**Fig. 3:**
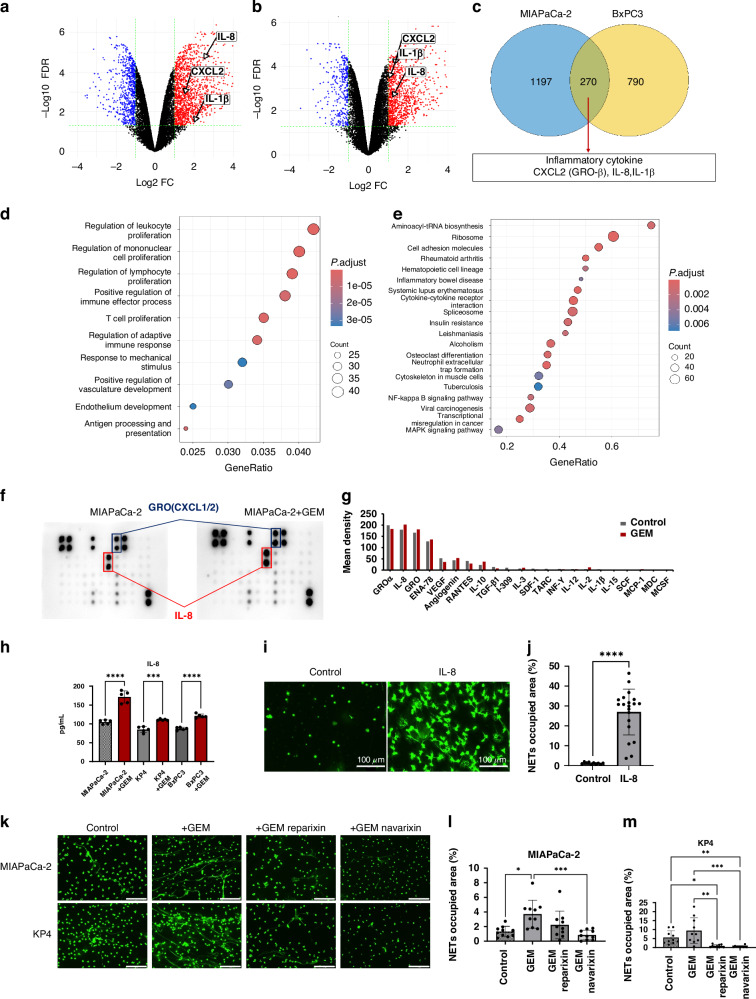
GEM-treated PDAC cells secrete more inflammatory cytokines. We analysed the public dataset GSE223303. This included data on gene expression 48 h after administration of gemcitabine to MIAPaCa-2 and BxPC3 cells. Volcano plot showing DEGs when gemcitabine was administered to MIAPaCa-2 (**a**) and BxPC3 (**b**) cells. **c** Venn diagram showing the upregulated genes in MIAPaCa-2 and BXPC3 cells. **d** GO analysis of the upregulated genes in MIAPaCa-2 cells. **e** GSEA visualises upregulated pathways using KEGG knowledge bases. **f** Image of the cytokine array using CM from MIAPaCa-2 cells with or without GEM. **g** Elevated levels of IL-8 and GRO were found in CM from GEM-treated MIAPaCa-2 cells. **h** An increase in IL-8 in the CM due to gemcitabine treatment was confirmed in all three cell lines using ELISA. **i** DNA staining image when IL-8 was added to neutrophils (scale bar: 100 μm). **j** Statistical evaluation of NETs induced by IL-8. **k** DNA staining images that tested whether CXCR inhibitors inhibited chemoNETosis (scale bar: 100 μm). Statistical evaluation of NETs in the MIAPaCa-2 (**l**) and KP4 (**m**) groups *P < 0.05; **P < 0.01; ***P < 0.001; ****P < 0.0001. Data are presented as the mean ± SD.

To elucidate the mechanism by which GEM-treated PDAC cells enhance NETs formation, we analysed the public dataset GSE223303. This dataset included information on gene expression changes in MIAPaCa-2 and BxPC3 cells following GEM treatment. DEGs were identified with and without GEM treatment (Fig. 3a, b). Among the upregulated genes common to both cell lines, we identified proinflammatory cytokines known to induce NETs, including CXCL2, IL-8 and IL-1β (Fig. 3c). GO and GSEA of upregulated genes in MIAPaCa-2 cells revealed the activation of pathways associated with leucocyte proliferation and NETs formation (Fig. 3d, e). Cytokine changes were then validated in CM using a cytokine assay. Consistent with the RNA sequencing results, IL-8 and CXCL1/2 levels were elevated in CM from GEM-treated MIAPaCa-2 cells (Fig. 3f, g). Focusing on IL-8, which has been reported to correlate positively with intratumoral NETs abundance in solid tumours [23], changes in IL-8 secretion were measured in various cell lines. GEM reproducibly induced IL-8 secretion in all the three cell lines tested (Fig. 3h). Furthermore, the addition of IL-8 to human neutrophils robustly induced NETs formation (Fig. 3i, j). IL-8 is known to mediate neutrophil chemotaxis and NETs formation via CXCR1/2 [15, 24]. Based on this, we hypothesised that CXCR1/2 inhibitors could suppress chemoNETosis. As expected, chemoNETosis was inhibited in vitro by both reparixin and navarixin, which have different affinities for CXCR1 and CXCR2 (Fig. 3k). Although the difference was not statistically significant, navarixin, which had a stronger inhibitory effect on CXCR2 [25], showed a greater NETs suppression effect (Fig. 3l, m). These findings suggest that GEM-treated PDAC cells secrete pro-inflammatory cytokines, such as IL-8, stimulating neutrophils to induce chemoNETosis. This phenomenon can be mitigated by using CXCR inhibitors.

### NETs induce further chemoresistance in PDAC cells

**Fig. 4:**
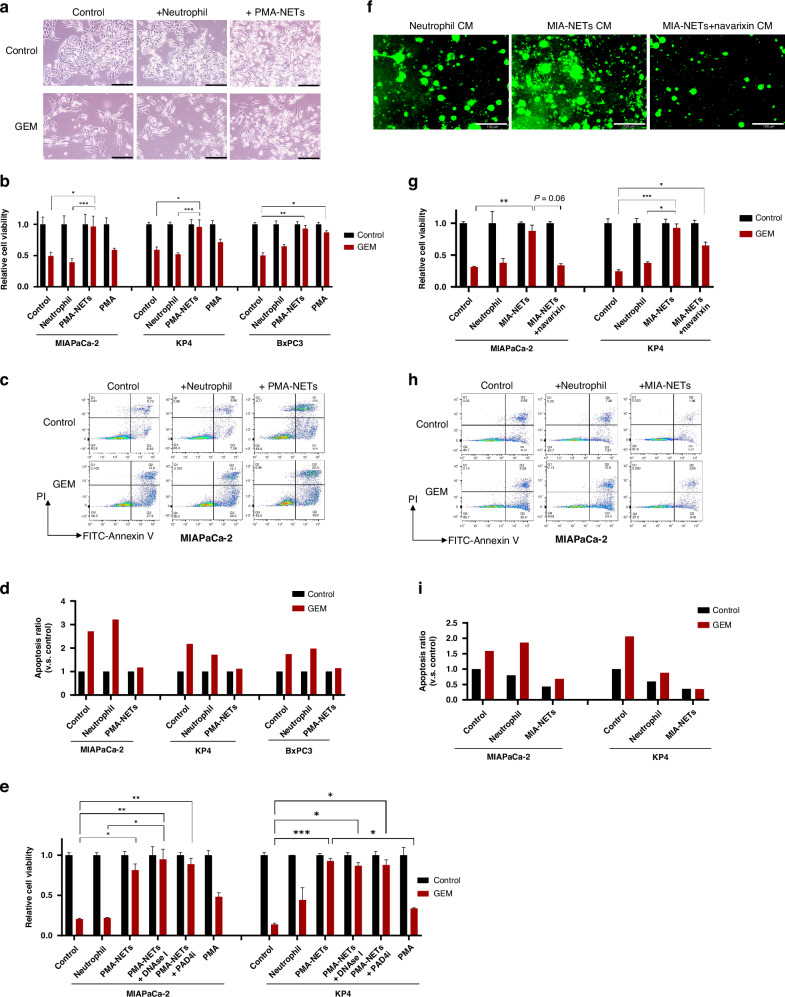
NETs induce chemoresistance in PDAC cells. **a** Representative image after 72 h of incubation with each CM ± GEM administered to MIAPaCa-2 (scale bar: 200 μm). **b** Relative cell viability by XTT assay in MIAPaCa-2, KP4 and BxPC3. **c** Apoptosis assay after 72 h of incubation with each CM ± GEM administered to MIAPaCa-2. **d** A graph showing the ratio of increased apoptosis with GEM administration, with no GEM administration as the standard for each condition. **e** Relative cell viability by XTT assay in MIAPaCa-2 and KP4. PMA-NETs-mediated chemoresistance could not be reversed by DNase I or PAD4i. **f** DNA staining images after collecting MIA-NETs CM (scale bar: 100 μm). **g** Relative cell viability by XTT assay in MIAPaCa-2 and KP4. **h** Apoptosis assay after 72 h of incubation with each CM ± GEM administered to MIAPaCa-2. **i** A graph showing the ratio of increased apoptosis with GEM administration, compared to neither GEM or CM administration. *P < 0.05; **P < 0.01; ***P < 0.001. Data were presented as means ± SD.

Having established that GEM-treated PDAC cells increase NETs formation, we next investigated how these NETs impact PDAC treatment. Using a previously established protocol for PMA-induced NETs collection [20], we treated PDAC cells with PMA-induced NETs conditioned medium (PMA-NETs CM). Compared to normal culture conditions or treatment with neutrophil CM, PMA-NETs CM reduced cell-cell adhesion in cancer cells. Interestingly, the cell density in the presence of PMA-NETs CM alone was lower than that in the control or neutrophil CM. However, it became the highest under GEM treatment, suggesting that NETs contribute to chemoresistance (Fig. 4a, b). Apoptosis assays revealed that NETs induced mild cytotoxicity in MIAPaCa-2 cells but abrogated the apoptosis-enhancing effect of GEM (Fig. 4c, d). In contrast, no cytotoxicity was observed in KP4 or BxPC3 cells following NETs treatment, but chemoresistance was consistently induced (Fig. S2A, B). Attempts to neutralise NETs using DNase I or PAD4i have been unsuccessful in reversing resistance to chemotherapy (Fig. 4e). To create more physiologically relevant conditions, we attempted to apply a co-culture system with neutrophils. However, chemoresistance was not induced in MIAPaCa-2 cells under co-culture conditions, potentially due to the limited lifespan of neutrophils in vitro (Fig. S2C). Therefore, we generated MIAPaCa-2-derived NETs conditioned medium (MIA-NETs CM) and NETs-suppressed CM using navarixin (Fig. 4f). Using these CM preparations, we found that MIA-NETs CM also induced chemoresistance, whereas navarixin treatment suppressed this resistance (Fig. 4g). Apoptosis assays demonstrated that MIA-NETs CM inhibited GEM-induced apoptosis, confirming the induction of chemoresistance. Unlike PMA-NETs, MIA-NETs did not exhibit cytotoxic effects (Fig. 4h, i and S2D). These findings suggest that the presence of NETs in PDAC promotes chemoresistance. Thus, targeting NETs formation may attenuate the resistance to chemotherapy.

### NETs upregulate anti-apoptotic proteins in PDAC cells

**Fig. 5:**
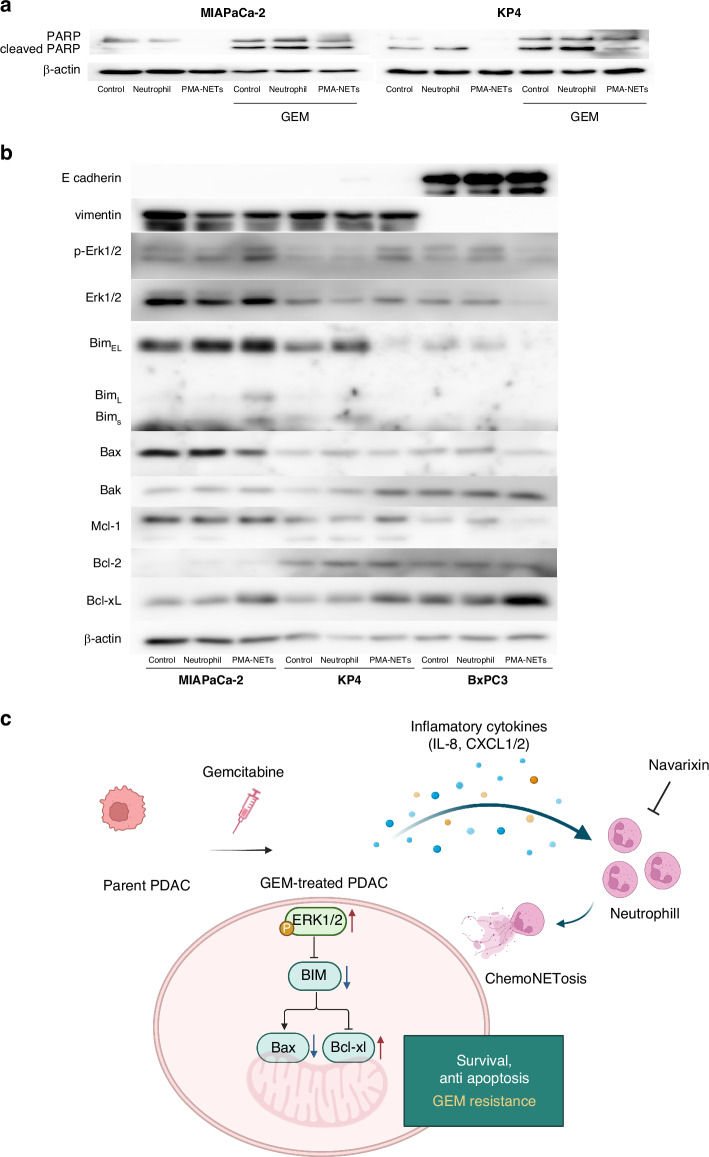
NETs upregulate anti-apoptotic proteins in PDAC cells. **a**, **b** Proteins were extracted from PDAC cells 72 h after treatment with each CM, and western blotting was performed. **c** Schematic representation of the hypothesis explored in this study. GEM-treated PDAC cells secreted inflammatory cytokines, that triggered chemoNETosis. NETs acted on PDAC cells to activate Erk1/2 signalling, upregulate Bcl-xL, thereby inducing chemoresistance.

To investigate how NETs act on PDAC cells to induce chemoresistance, we performed western blot analysis. Consistent with the apoptosis assay results, addition of PMA-NETs reduced the levels of cleaved poly (ADP-ribose) polymerase (Fig. 5a). Previous studies have reported that NETs induce EMT in PDAC [14], and chemoNETosis is associated with EMT-dependent chemoresistance mediated by TGF-β signalling in metastatic breast cancer [20]. However, as MIAPaCa-2 and KP4 are mesenchymal tumours, we hypothesised that mechanisms beyond EMT might contribute to the chemoresistance. Further analysis of apoptosis-regulating proteins revealed that NETs treatment increased Bcl-xL and decreased Bax (Fig. 5b). Additionally, phosphorylation of Erk1/2, a signalling molecule upstream of the Bcl-2 family [26], was elevated following NETs treatment. As expected, EMT markers were not detected by western blotting in mesenchymal-type cell lines. In summary, GEM-treated PDAC cells secrete inflammatory cytokines such as IL-8 that trigger chemoNETosis. The resulting NETs acted on PDAC cells to activate Erk1/2 signalling, upregulate Bcl-xL and downregulate Bax, thereby inducing chemoresistance (Fig. 5c).

### Combination anti-NETs therapy and GEM has synergistic effects on PDAC

**Fig. 6:**
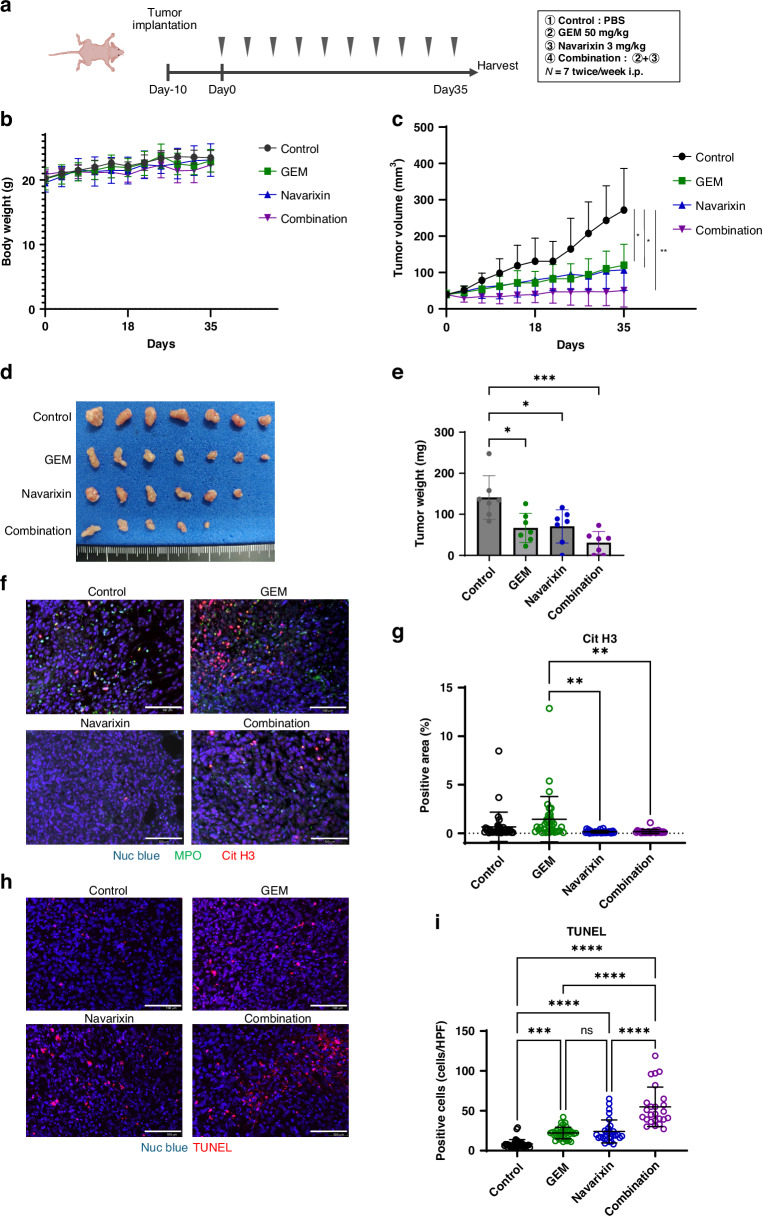
Combination anti-NETs therapy and GEM has synergistic effects on PDAC. **a** In vivo experiment protocol for mouse subcutaneous tumour model (7 mice/group). Mice were injected intraperitoneally twice a week for 5 weeks with a 100-µL volume of PBS or GEM (50 mg/kg) or navarixin (3 mg/kg) or GEM and navarixin. **b** Graph showing changes in body weight during treatment experiment. **c** Tumour growth curves for non-treated control (black line) and GEM-treated (green line), Navarixin-treated (blue line) and Combination-treated (purple line) mice. **d** Photographs of tumours in the four groups. The combination therapy resulted in complete remission (CR) in 2 of 7 mice. **e** Graph showing the weight of the tumours in the four groups. **f** Immunofluorescence staining of Nuc blue (blue), MPO (green) and CitH3 (red) in the tumour of the four groups (scale bar: 100 μm). **g** Statistical evaluation of NETs based on CitH3-positive area. **h** TdT-mediated dUTP Nick End Labelling (TUNEL) assay in the tumour of the four groups (scale bar: 100 μm). **i** Statistical evaluation of TUNEL-positive cells per high-power field (HPF). *P < 0.05; **P < 0.01; ***P < 0.001; ****P < 0.0001; ns no significance. Data were presented as means ± SD.

Based on our findings, we hypothesised that chemoNETosis could serve as a novel therapeutic target for PDAC treatment. The efficacy of combining GEM with navarixin, which inhibits chemoNETosis in vitro, was investigated in subcutaneous pancreatic tumours (Fig. 6a). This combination therapy was safely administered without significant weight loss (Fig. 6b). After 5 weeks of treatment, both GEM and navarixin monotherapy demonstrated modest anti-tumour effects, whereas the combination therapy achieved the most significant tumour regression (Fig. 6c). Remarkably, combination therapy resulted in complete remission in two of the seven mice (Fig. 6d). Tumour weight was also the lowest in the combination therapy group, indicating superior efficacy (Fig. 6e). Immunohistochemical analysis of the harvested TME revealed the highest levels of tumour-infiltrating neutrophils and intratumoral NETs in the GEM-treated group. These levels were significantly suppressed in both the navarixin and combination therapy groups (Fig. 6f, g and Fig. S3). Furthermore, apoptotic cells within the tumour tissue were most abundant in the combination therapy group (Fig. 6h, i). These results suggest that navarixin effectively inhibits chemoNETosis induced in vivo, and that targeting NETs could enhance the efficacy of conventional chemotherapy, representing a promising new therapeutic approach.

## Discussion

This study provides novel insights into the mechanisms of chemoNETosis in PDAC. Inflammatory cytokines upregulation leads to NETs formation, which in turn promotes chemoresistance by activating the Erk signalling pathway and modulating the Bcl-2 family. Additionally, chemoresistant PDAC cells were found to enhance NETs formation, suggesting that NETs create a vicious cycle that exacerbates chemoresistance in PDAC. These findings elucidate the previously unclarified mechanisms of chemoresistance in PDAC and highlight chemoNETosis as a potential therapeutic target. Mousset et al. recently reported chemoNETosis in metastatic breast cancer; They revealed that chemotherapy-treated cancer cells secrete IL-1β, which triggers NETs formation. Within NETs, integrin αvβ1 traps latent TGF-β. Matrix metalloproteinase 9 then cleaves and activates the trapped latent TGF-β. This activation induces cancer cells to undergo EMT, a process associated with chemoresistance. NETs were absent in tumours without chemotherapy treatment, and NETs formation occurred only in response to chemotherapy [20]. In contrast, our study showed that PDAC cell-derived CM could independently induce baseline NETs formation, which was further augmented by GEM treatment. These differences may reflect variations in tumour characteristics or the microenvironment [27]. Nevertheless, the observed enhancement of NETs formation in response to chemotherapy represents an additional therapeutic target for PDAC treatment.

Our GSEA analysis suggested that NF-κB signalling is activated following gemcitabine treatment (Fig. 3e), potentially contributing to the upregulation of IL-8. Previous studies have reported that gemcitabine induces ROS production via Nox, which activates NF-κB and STAT3 signalling pathways, leading to increased IL-8 expression [28–31]. These findings support a possible mechanism whereby gemcitabine triggers IL-8 secretion through ROS-mediated activation of NF-κB/STAT3 signalling.

NETs have been reported to exert both pro-tumorigenic and anti-tumorigenic effects. While this study demonstrated the pro-malignant role of NETs in inducing chemoresistance, prior studies have suggested that NETs induced by chemotherapy can exhibit anti-tumour effects, such as reactive oxygen species (ROS)-mediated cancer cell damage in colorectal cancer [32]. In addition, in patients with high-grade ovarian cancer, an increase in NETs-associated molecules in the ascites fluid was correlated with a favourable prognosis [33], or in melanoma, co-culturing of NETs and melanoma cells had a cytotoxic effect, resulting in necrosis [34]. In our study, PMA-induced NETs exhibited cytotoxic effects in MIAPaCa-2 cells; however, this phenomenon was not observed in NETs induced by MIA-CM. Furthermore, the other cell lines did not exhibit cytotoxicity when exposed to PMA-induced NETs. These results suggest that the functional roles of NETs may vary depending on their composition, density within the TME, or cancer cell sensitivity, which may differ across cancer types [27]. Notably, the tumour-promoting or anti-tumorigenic effects of NETs may also depend on the levels of ROS produced [35]. High ROS levels can induce oxidative damage in cancer cells and exert anti-tumour effects. In contrast, lower ROS levels may support tumour growth and survival. This balance could explain the variable responses observed in different cell lines in our study.

Although we demonstrated that NETs affect PDAC cells, the exact components of NETs that contribute to chemoresistance in PDAC remain unclear. Previous studies have highlighted various components of NETs that contribute to their multifaceted effects on cancer progression [12]. DNA strands, as the structural elements of NETs, play a pivotal role in their stability and interactions with tumour cells [36–38]. Neutrophil elastase (NE) and matrix metalloproteinase-9 (MMP-9), both proteolytic enzymes, have been shown to degrade extracellular matrix components, facilitating tumour invasion and metastasis [39–42]. Additionally, cathepsin G, another serine protease abundant in NETs, has been reported to enhance the invasive capabilities of hepatocellular carcinoma cells [43]. Moreover, PD-L1, which can be enriched in NETs, has been suggested to contribute to the formation of an immunosuppressive TME by inhibiting T cell activity, thereby facilitating tumour immune evasion [44]. On the tumour side, receptors, such as toll-like receptors and CCDC25, are instrumental in mediating cancer cell responses to NETs, including enhanced migration and survival [38, 42, 45]. As the induction of chemoresistance by NETs revealed in this study may be attributed to any of these factors or a combination thereof. A CXCR inhibitor that broadly suppress neutrophil infiltration and NETs generation was applied and effectively suppress NETs.

Other NETs inhibitors, including PAD4i and DNase I, were employed in this study, but failed to completely abrogate chemoresistance in vitro (Fig. 4e). This might be attributed to the inability of PAD4i to completely suppress robust NETs formation induced by PMA and the limited capacity of DNase I to inhibit the formation of NETs despite degrading their DNA scaffolds [46]. These findings underscore the need for novel therapeutic strategies targeting specific components or signalling pathways of NETs to achieve more effective inhibition.

This study identified CXCR1/2 as a potential target for reversing chemoresistance in PDAC cells. CXCR2 inhibition was previously shown to augments immunotherapy [47]. In a phase II trial evaluating the combination of navarixin and pembrolizumab in patients with solid tumours after standard treatments, but failed to demonstrate efficacy [48]. Thus, the selection of effective patient populations is essential when considering its combination with chemotherapy. Simple biomarkers, such as the neutrophil-to-lymphocyte ratio [6, 49] or plasma NETs levels [50, 51], have been reported to correlate with prognosis, suggesting their potential utility in patient selection. Navarixin effectively inhibits neutrophil recruitment via CXCR1/2 blockade. However, its clinical use may be associated with adverse effects such as neutropenia and increased risk of infection. However, in previous clinical trials [48, 52], neutropenia was generally manageable and did not lead to a significant increase in serious infections, suggesting that such risks can be mitigated with appropriate monitoring. Another promising approach is genetic analysis at the time of diagnosis. For instance, mutations in *TP53* in PDAC have been associated with increased neutrophil infiltration, indicating that genetic profiling may assist in identifying patients who are likely to benefit from NETs-targeted therapies [9].

Interactions between chemoNETosis and other cellular components of the TME may also be important. In pancreatic cancer, pancreatic stellate cells (PSCs) are known to be associated with chemoresistance [53], and NETs have been shown to activate PSCs [54]. Therefore, NETs may also contribute to chemoresitance by influencing PSCs. In this way NETs not only exert direct effects on tumours but also influence tumour progression through their interactions with other TME components [27]. For example, activated platelets bound to intrahepatic cholangiocarcinoma cells have been reported to induce NETs formation, thereby promoting metastasis [55]. Similarly, regulatory T cells may be enhanced by the immunosuppressive environment created by NETs in non-alcoholic steatohepatitis [56]. Additionally, NETs components have been reported to affect endothelial cells and promote vascular dysfunction [57] and lymphatic permeability [58]. These interactions represent critical factors to consider when evaluating the clinical efficacy of NETs-targeted therapies and underscore the need for further research in this area.

Overcoming chemoresistance remains a significant challenge in PDAC treatment. The results of this study suggest that targeting NETs may be a breakthrough in addressing this issue. Thus, it is imperative to develop therapeutic strategies that selectively inhibit the pro-tumorigenic effects of NETs. At the same time, the physiological functions of neutrophils must be preserved to ensure both efficacy and safety in clinical applications.

## Supplementary information

Supplemental Table and Figures

Supplementary Methods

## Data Availability

The data analysed in this study were obtained from GEO at GSE223303. The other analysed datasets generated during this study are available from the corresponding author upon reasonable request.
