# Supplementary material for: Gemcitabine-induced neutrophil extracellular traps via interleukin-8-CXCR1/2 pathway promote chemoresistance in pancreatic cancer
Source: Br J Cancer. 2025 Sep 25;133(11):1640–51. doi: 10.1038/s41416-025-03192-1 (PMC12644870; doi:10.1038/s41416-025-03192-1)
Supplement: Supplementary file 1 — Supplemental Table and Figures [file 41416_2025_3192_MOESM1_ESM.pdf]

Table S1

		Grade I (n=7)	Grade ≥ IIb (n=12)	p
**Age**		72.4±5.0*	68.8±7.2*	0.24
**SEX**	**M**	4 (57.1%)	5 (41.7%)	0.51
	**F**	3 (42.9%)	7 (58.3%)	
**Tumor size (mm)**		28.3 ± 6.9*	28.7 ± 8.2*	0.92
**f Stage**	**II**	7 (100%)	8 (66.7%)	0.086
	**III**	0 (0%)	4 (33.3%)	
**NLR (pre-ope)**		2.47 ± 1.22*	2.06 ± 0.98*	0.49
**Resectability**	**R**	2 (28.6%)	4 (33.3%)	0.28
	**BR**	5 (71.4%)	5 (41.7%)	
	**UR-LA**	0 (0%)	3 (25%)	
**Curability**	**R0**	2 (28.6%)	12 (100%)	0.00065
	**R1**	5 (71.4%)	0 (0%)	
**Outcome**	**alive**	2 (28.6%)	4 (33.3%)	0.83
	**dead**	5 (71.4%)	8 (66.7%)	

Table S1. Patient background for clinical sample analysis. * means ± SD

Figure S1

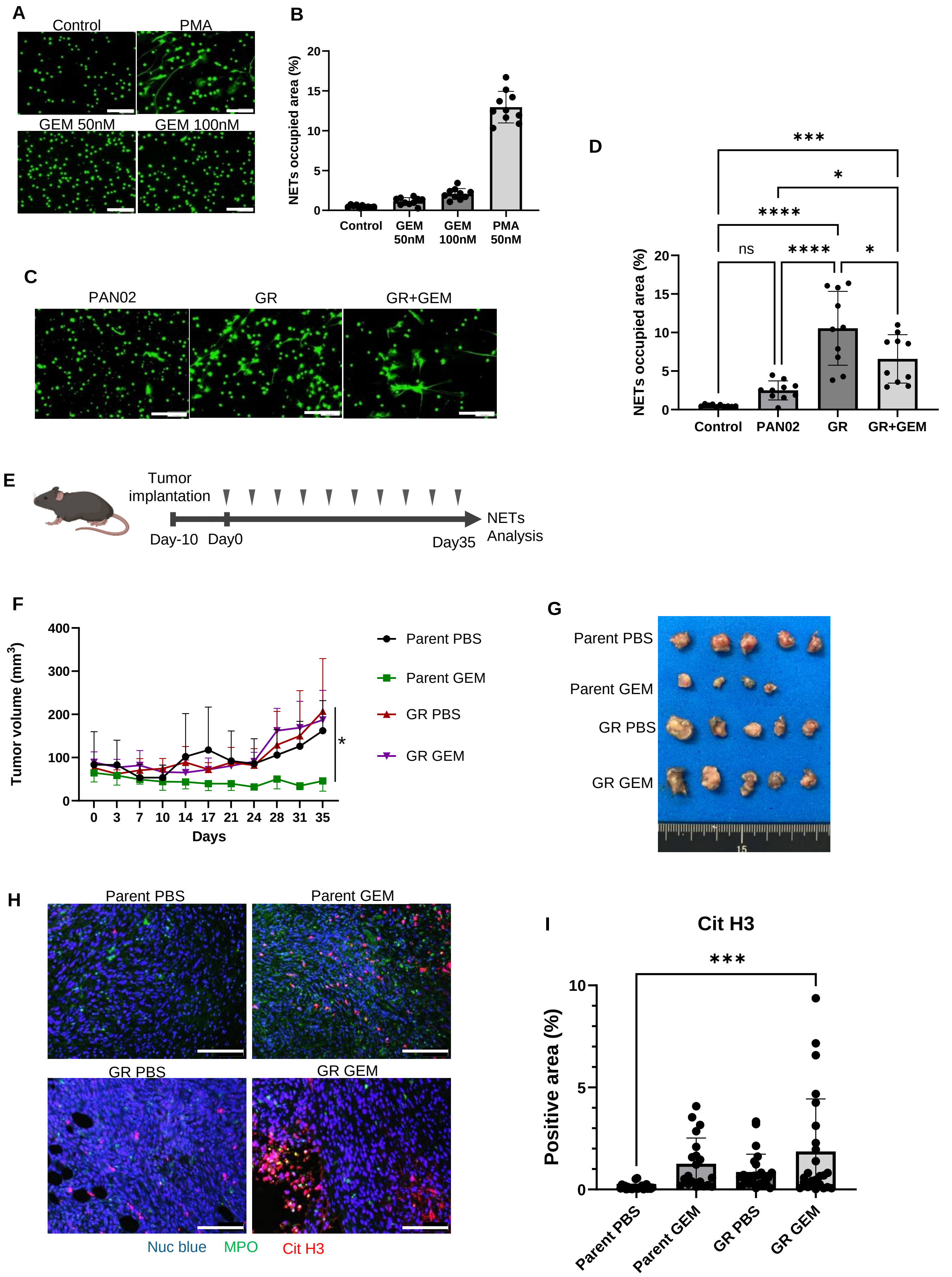

Figure S1. Gemcitabine-treated mouse PDAC cells induce ChemoNETosis

A DNA staining image when adding Gemcitabine or PMA (scale bar: 100 μm). **B** Statistical evaluation of NETs by gemcitabine stimulation based on extracellular DNA occupied area. **C** DNA staining image when adding CM from PAN02 (scale bar: 100 μm). **D** Statistical evaluation of NETs by CM from PAN02. CM from GR PAN02 cells causes strong NETs. **E** In vivo experiment protocol for mouse subcutaneous tumor model (3 mice/group). Mice were injected intraperitoneally twice a week for 5 weeks with a 100-μL volume of PBS or GEM (100 mg/kg). **F** Tumor growth curves for Parent-PBS treated (black line), Parent-GEM treated (green line), GR-PBS treated (red line) and GR-GEM-treated (purple line) mice. **G** Photographs of tumors in the four groups. **H** Immunofluorescence staining of Nuc blue (blue), MPO (green) and CitH3 (red) in the tumor of each condition (scale bar: 100 μm). **I** Statistical evaluation of NETs in the four groups based on CitH3-positive area. *P < 0.05; **P < 0.01; ***P < 0.001; ****P < 0.0001; ns, no significance. Data were presented as means ± SD

Figure S2

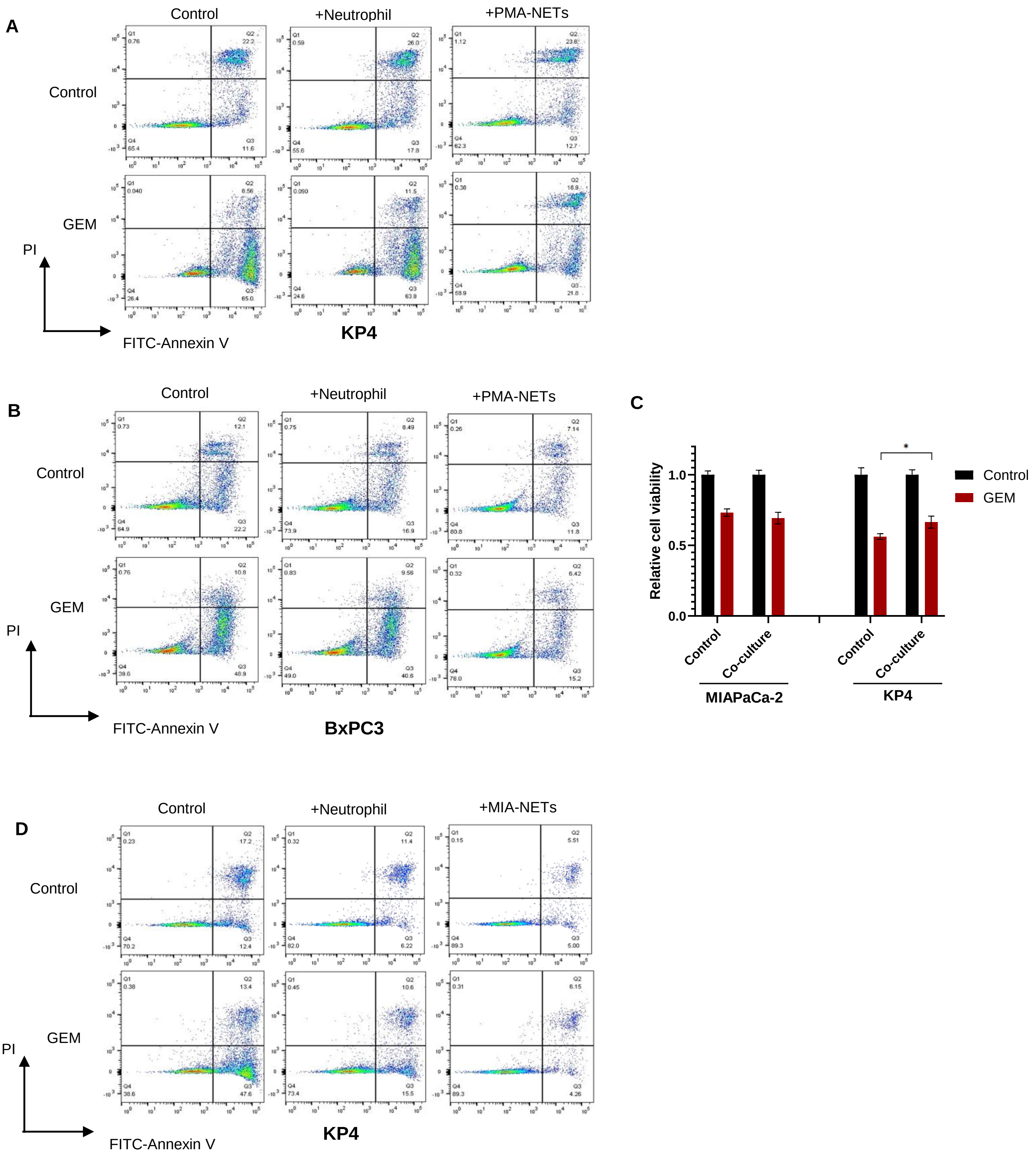

Figure S2. Apoptosis assay in KP4 and BxPC3
A,B Apoptosis assay after 72 h of incubation with PMA-NETs CM ± GEM administered to KP4 (**A**) and BxPC3 (B). **C** Relative cell viability assessed by XTT assay in MIAPaCa-2 and KP4 cells with or without co-culture with neutrophils. **D** Apoptosis assay after 72 h of incubation with MIA-NETs CM ± GEM administered to KP4. *$P < 0.05$. Data were presented as means ± SD.

Figure S3

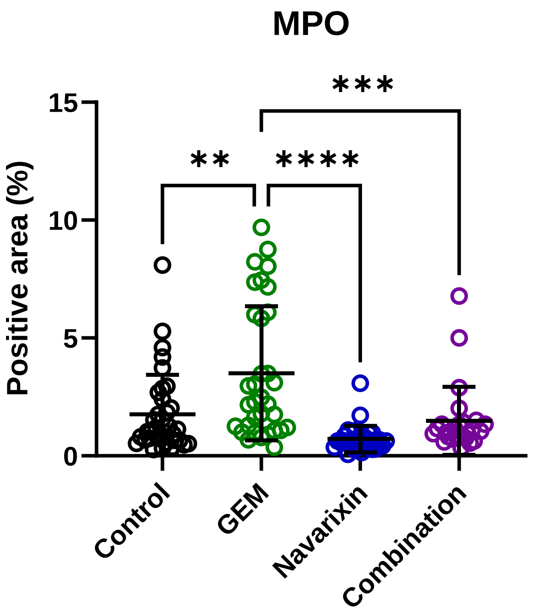

Figure S3. Statistical evaluation of neutrophil infiltration **based on MPO-positive area in mouse subcutaneous tumor model**
*P < 0.05; **P < 0.01; ***P < 0.001; ****P < 0.0001. Data were presented as means ± SD

Figure S4

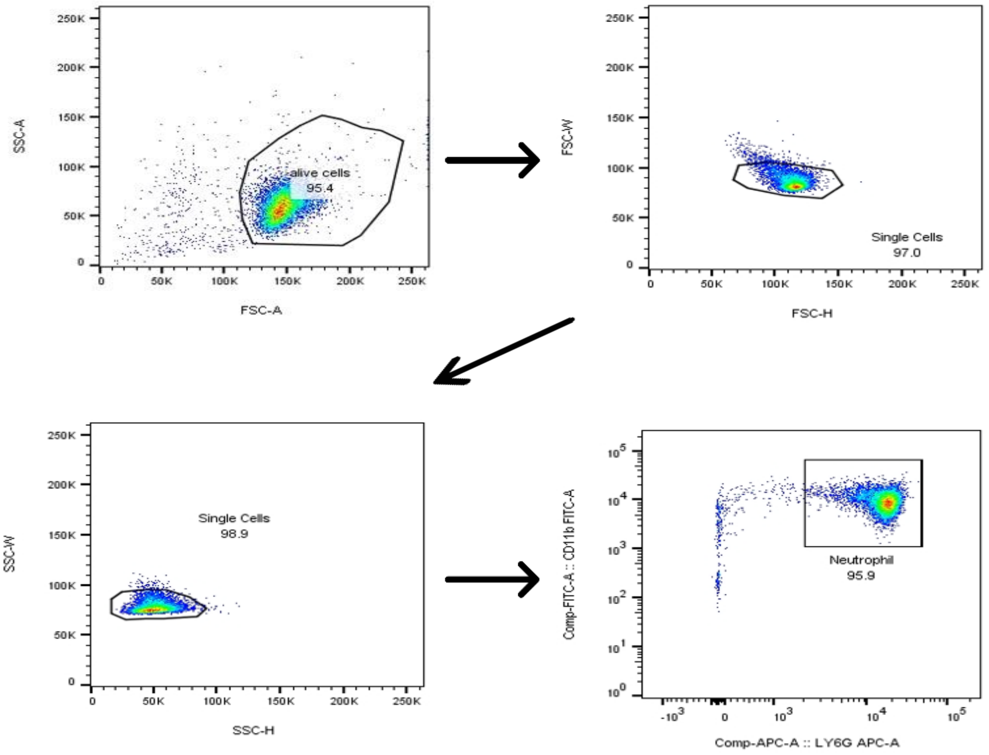

Figure S4. Mouse neutrophils were purified using Neutrophil Isolation kit
Neutrophil purity flow. Among the cells gated as alive and singlets, 95.9% were identified as CD11b/Ly6G-positive.
