# Supplementary material for: Gemcitabine-induced neutrophil extracellular traps via interleukin-8-CXCR1/2 pathway promote chemoresistance in pancreatic cancer
Source: Br J Cancer. 2025 Sep 25;133(11):1640–51. doi: 10.1038/s41416-025-03192-1 (PMC12644870; doi:10.1038/s41416-025-03192-1)
Supplement: Supplementary file 2 — Supplementary Methods [file 41416_2025_3192_MOESM2_ESM.pdf]

Supplementary Methods

Cell culture and reagents

MIAPaCa-2, BxPC3, KP4 and PAN02 were cultured in DMEM or RPMI-1640 supplemented with 10% fetal bovine serum (FBS), 100 U/mL penicillin, and 100 µg/mL streptomycin (#168-23191, FUJIFILM). The cultures were maintained at 37 °C in a humidified incubator with 20% O_2 and 5% CO_2. GR PAN02 cells were generated by sequential exposure to GEM (Gemzar; Eli Lilly, Japan) at concentrations adjusted monthly based on the 50% inhibitory concentration (IC50) values determined by XTT assays over a 3-month period. GEM was used at 100 nM for MIAPaCa-2 and 10 nM for other cell lines in the in vitro study; PMA (#P1585, Sigma-Aldrich) was used at 20 nM for human neutrophils and 50 nM for mouse neutrophils; PAD4i, GSK484 at 1 µM (#17488, Cayman); DNase I was used at 1.5U/mL (#10104159001, Roche); CXCRi navarixin (#S8506, Selleck) and reparixin (#S8640, Selleck) were both used at 10 nM; IL-8 was used at 100 ng/mL (#I1645, Sigma-Aldrich).

Immunofluorescence staining

Paraffin-embedded tissue samples were deparaffinised and rehydrated. Non-specific

binding was blocked using 10% donkey serum (#ab7475, Abcam) for 30 min at room temperature. To detect neutrophils and NETs, the samples were incubated overnight at 4 °C with anti-MPO antibody (1:1000, #AF3667, RSD) and anti-CitH3 antibody (1:1000, #ab281584, Abcam), respectively[19]. The tissues were then incubated with secondary antibodies Alexa Fluor 488 (1:1000, #ab150129, Abcam) and Alexa Fluor 647 (1:1000, #ab150075, Abcam) for 1 h at room temperature. After the samples were enclosed in NucBlue™ (#P36981, Thermo Fisher), images were obtained using an IX83 microscope. NETs in tissue samples were determined as the percentage of cells positive for CitH3. For NETs quantification in tissue samples, NETs were counted in at least five HPFs at 200× magnification per section and two sections per sample were evaluated. The images were analyzed using CellSens Dimension Desktop software ver. 4.1 (Olympus, Tokyo, Japan).

Human neutrophil isolation

Neutrophils were isolated from healthy volunteers. Whole blood was collected by venipuncture into blood collection tubes coated with EDTA 2 K (#365900; Becton, Dickinson and Company). Then, 5 mL of whole blood was layered over 5 mL of Polymorphprep (#114683, Abbott Diagnostics Technologies AS) in a 15-mL tube and

centrifuged at 500 g for 30 min at room temperature. The lower leukocyte band containing neutrophils was collected, followed by exclusion of erythrocytes by reacting with RBC Lysis Buffer (#420302, BioLegend) for 2 min and washing with phosphate-buffered saline. Finally, the cells were resuspended in the RPMI medium without FBS.

Mouse neutrophil isolation

Neutrophils were isolated from the femoral bone marrow of 6-week-old female C57BL/6 J mice (CLEA Japan, Tokyo, Japan) using a Neutrophil Isolation Kit (#130-097-658, Miltenyi Biotec) according to the manufacturer’s instructions. To evaluate the accuracy of isolated neutrophils, flow cytometry analysis confirmed that 95.9% of the cells were positive for both FITC anti-CD11b (#101206, BioLegend) and APC anti-Ly6G (#127614, BioLegend) (Figure S4).

Conditioned media preparation

Conditioned media (CM) from the PDAC cell line was collected after 48 h of incubation in serum-free RPMI-1640 at 100% confluence. After centrifugation, the supernatant was used as the cancer CM and stored at -80°C.

Cytokine array

To detect 42 human cytokines, CM was assayed using a human cytokine antibody array membrane (#ab133997, Abcam) according to the manufacturer's instructions.

Preparation of NETs

Neutrophils (5×10^6 cells/dish) were seeded in cell culture dishes (100 × 20 mm, #353003, FALCON). For PMA-NETs, neutrophils were stimulated with PMA with or without DNase I or GSK484 for 6 h; for MIA-NETs, neutrophils were stimulated with MIA-CM with or without navarixin overnight. Neutrophil CM was prepared by culturing neutrophils without adding any reagents under each condition. Subsequently, the supernatant was slowly aspirated and centrifuged at 500 g for 5 min to remove cell debris, which was then stored at -80 °C.

Western blotting

Protein was extracted from whole cells after 72 h with or without CM. The concentrations of extracted proteins were measured using standard protocols. Cells were lysed using a cell lysis buffer (50 mmol/L Tris-HCl (pH 7.4), 30 mmol/L NaCl, and 1% TritonX-100) containing cOmplete Mini (#11836153001, Roche) and PhosSTOP™

(#12352204, Roche) as protease inhibitors and sonicated. Equal amounts of protein (25 µg/lane) were loaded on sodium dodecyl sulfate-polyacrylamide gel and transferred onto Amersham Hybond P 0.45 PVDF (#10600023, GE Healthcare) according to the manufacturer's protocol. The membranes were incubated with Blocking One (#03953-95, Nacalai, Kyoto, Japan) or Blocking One-P (#05999-84, Nacalai, Kyoto, Japan) for 30 min at room temperature and incubated with primary antibodies overnight at 4 °C. The membranes were then washed three times for 5 min in Tris-buffered saline Tween 20 (TBST, 10 mM Tris-HCL (pH 7.5), 500 mM NaCl, and 0.1% Tween 20), followed by incubation with secondary antibodies for 1 h at room temperature, and washed three times with TBST. The ECL Prime Western Blotting Detection Reagent (#RPN2232, GE Healthcare) was used to detect the peroxidase activity of the secondary antibodies. The membranes were probed with β-actin as a loading control. The primary antibodies used were as follows: β-actin (#A5441, Sigma-Aldrich), poly (ADP-ribose) polymerase (PARP) (#9542, Cell Signaling Technology, Danvers, MA, USA), Bcl-2 (#4223, Cell Signaling Technology), Bcl-xL (#2764, Cell Signaling Technology), Mcl-1 (#5453, Cell Signaling Technology), Bak (#12105, Cell Signaling Technology), Bax (#5023, Cell Signaling Technology), Bim (#2973, Cell Signaling Technology), Erk1/2 (#4695, Cell Signaling Technology), phosphor-Erk1/2 (#4370, Cell Signaling Technology), E-

cadherin (#3195, Cell Signaling Technology), and vimentin (#5741, Cell Signaling Technology). All primary antibodies were used at a 1:1000 dilution. The secondary antibodies used were anti-mouse IgG, HRP-linked Whole Antibody Sheep (#NA931, GE Healthcare, Buckinghamshire, England, UK) at a 1:2500 dilution and anti-rabbit IgG, HRP-Linked Whole Antibody Donkey (#NA934, GE Healthcare) at a 1:5000 dilution.

In vivo apoptosis evaluation

Apoptosis in tumor tissues was evaluated using the Click-iT™ Plus TUNEL Assay Kit for In Situ Apoptosis Detection Alexa Fluor™ 647 (# AC10619, Thermo Fisher), with minor modifications to the manufacturer’s protocol. Specifically, after TUNEL staining, the samples were enclosed in ProLong™ Glass Antifade Mountant with NucBlue™ Stain to enable nuclear visualization, and images were acquired using IX83 microscope. For quantification, TUNEL-positive cells were counted in five HPFs at 200× magnification per section. Additionally, to ensure accurate cell identification, signals with an individual positive area <10 μm^2 were excluded from the analysis as artifacts.

Code availability

The computer code used for data analysis is available from the corresponding author upon reasonable request.
